# Somatic Copy Number Alterations and Associated Genes in Clear-Cell Renal-Cell Carcinoma in Brazilian Patients

**DOI:** 10.3390/ijms22052265

**Published:** 2021-02-25

**Authors:** Flávia Gonçalves Fernandes, Henrique Cesar Santejo Silveira, João Neif Antonio Júnior, Rosana Antunes da Silveira, Luis Eduardo Zucca, Flavio Mavignier Cárcano, André Octavio Nicolau Sanches, Luciano Neder, Cristovam Scapulatempo-Neto, Sergio Vicente Serrano, Eric Jonasch, Rui Manuel Reis, Adriane Feijó Evangelista

**Affiliations:** 1 Molecular Oncology Research Center, Barretos Cancer Hospital, Barretos 14784-400, Brazil; fernandesflaviag@gmail.com (F.G.F.); henriquecssilveira@gmail.com (H.C.S.S.); radsilveira@gmail.com (R.A.d.S.); 2 Department of Medical Oncology, Barretos Cancer Hospital, Barretos 14784-400, Brazil; neifjr@msn.com (J.N.A.J.); eduardozucca@hotmail.com (L.E.Z.); carcano.fm@gmail.com (F.M.C.); ansanches@hotmail.com (A.O.N.S.); svserrano1@gmail.com (S.V.S.); 3 Barretos School of Health Sciences Dr Paulo Prata-FACISB, Barretos 14785-002, Brazil; 4 Department of Pathology, Barretos Cancer Hospital, Barretos 14784-400, Brazil; neder@fmrp.usp.br (L.N.); cristovamscapula@gmail.com (C.S.-N.); 5 Department of Genitourinary Medical Oncology, Division of Cancer Medicine, The University of Texas MD Anderson Cancer Center, Houston, TX 77030, USA; EJonasch@mdanderson.org; 6 Life and Health Sci Research Institute (ICVS), Medical School, University of Minho, 4710-057 Braga, Portugal; 7 ICVS/3B’s-PT Government Associate Laboratory, 4710-057 Braga/Guimarães, Portugal

**Keywords:** copy number aberrations, clear-cell renal-cell carcinoma, bioinformatics, Brazilian population

## Abstract

Somatic copy number aberrations (CNAs) have been associated with clear-cell renal carcinoma (ccRCC) pathogenesis and are a potential source of new diagnostic, prognostic and therapeutic biomarkers. Recurrent CNAs include loss of chromosome arms 3p, 14q, 9p, and gains of 5q and 8q. Some of these regional CNAs are suspected of altering gene expression and could influence clinical outcomes. Despite many studies of CNAs in RCC, there are currently no descriptions of genomic copy number alterations in a Brazilian ccRCC cohort. This study was designed to evaluate the chromosomal profile of CNAs in Brazilian ccRCC tumors and explore clinical associations. A total of 92 ccRCC Brazilian patients that underwent nephrectomy at Barretos Cancer Hospital were analyzed for CNAs by array comparative genomic hybridization. Most patients in the cohort had early-stage localized disease. The most significant alterations were loss of 3p (87.3%), 14q (35.8%), 6q (29.3%), 9p (28.6%) and 10q (25.0%), and gains of 5q (59.7%), 7p (29.3%) and 16q (20.6%). Bioinformatics analysis revealed 19 genes mapping to CNA significant regions, including *SETD2*, *BAP1*, *FLT4*, *PTEN*, *FGFR4* and *NSD1*. Moreover, gain of 5q34-q35.3 (*FLT4* and *NSD1*) and loss of 6q23.2-q23.3 (*MYB*) and 9p21.3 (*MLLT3*) had gene expression levels that correlated with TCGA data and was also associated with advanced disease features, such as larger tumors, Fuhrman 3, metastasis at diagnosis and death. The loss of region 14q22.1 which encompasses the *NIN* gene was associated with poor overall survival. Overall, this study provides the first CNA landscape of Brazilian patients and pinpoints genomic regions and specific genes worthy of more detailed investigations. Our results highlight important genes that are associated with copy number changes involving large chromosomal regions that are potentially related to ccRCC tumorigenesis and disease biology for future clinical investigations.

## 1. Introduction

Renal-cell carcinoma (RCC) comprises a complex set of diseases with different histopathological subtypes, clinical courses, and therapeutic responses [1,2,3,4]. Kidney cancer represented 2–3% of all cancers, affecting over 403,000 new individuals and accounted for around 175,000 cancer-related deaths worldwide in 2018 [5]. The 5-year survival rate for localized kidney cancer is up to 90%, but this rate drops dramatically for patients with distant metastasis (11.9%), accounting for 30% of the advanced disease diagnosed at presentation [5,6]. The clear-cell subtype is the most common histological subtype of renal-cell carcinoma (70–85%) and accounts for the most RCC-specific deaths [7]. In Brazil, the estimated number for 2018 was 10,688 new cases and 4084 deaths [5]. At Barretos Cancer Hospital, which receives patients from all regions in Brazil, urologic tumors accounted for 12.7% of all cancer cases in 2017, and over 150 new cases of kidney cancer were diagnosed in the same year (1.4% of all cases). More than half of the patients were diagnosed at stages II and IV, with a 5-year survival rate of 56% and 10%, respectively (https://infogram.com/rhc_hcb).

Recurrent chromosomal abnormalities and copy number imbalances are related to clear-cell renal-cell carcinoma (ccRCC) tumorigenesis [8]. Most of the ccRCC copy number alterations (CNAs) consist of extensive chromosomal losses or gains that can involve multiple genes, in which changes of gene dosage may alter the levels of gene’s expression [9,10,11,12,13]. The impact of CNAs has been recently associated with changes in the metabolic activity since copy number losses of *KEAP1* are involved in the transcriptional control of glutathione metabolism genes [14]. The comparison of the CNAs profile with the germline alterations in single cells from *VHL/PBRM1*-negative ccRCCs showed higher amplification of these alterations, suggesting an important role of CNAs in the progression of these tumors [15]. Indeed, recurrent CNAs have been shown to interfere with some of the affected gene’s functions [11,12,13], but there is presently no established association with specific genes affected by CNAs and the ccRCC tumor biology or disease progression. Importantly, CNAs may constitute useful diagnostic, therapeutic and prognostic biomarkers [16,17].

Loss of chromosome 3p is the major cytogenetic hallmark of ccRCC [18,19]. The loss or inactivation of the von Hippel–Lindau (*VHL*) tumor-suppressor gene at 3p25.3 is considered the crucial step in ccRCC carcinogenesis; however, other alterations are also required [4,10]. The Cancer Genome Atlas (TCGA) consortium has characterized and made publicly available over 20,000 primary cancer and matched normal samples from 33 cancer types, including ccRCC and other kidney cancer types [10]. The TCGA datasets include genomic, transcriptomic, and protein level biological information that has validated the most frequent alterations in ccRCC, providing new insights about potential therapeutic targets, such as PI(3)K/AKT pathway and SWI/SNF chromatin remodeling complex [10]. Losses of chromosomes 14q, 9p and deletions on 6q and 8p are also reported and have been described to be correlated with advanced tumors and worse patient survival [17,20,21,22]. Gain of chromosome 5q is the only CNA associated with better patient survival [17]. Understanding the role of discrete CNAs and its impacts on gene dosage are essential to better characterize the ccRCC pathogenesis, and may be helpful in future clinical settings.

Despite multiples studies on RCC CNAs, there are no current reports of chromosomal copy number alterations in a Brazilian ccRCC cohort and their possible clinicopathological correlations. Also, data regarding the relationship between CNA and molecular aspects of RCC in Brazil are still scarce [23]. Therefore, this study aimed to evaluate the CNA profile in a series of Brazilian ccRCC patients and explore putative clinicopathological associations related to CNA-driven genes’ expression as well as describe new potential molecular features and possible molecular targets that could be worthy for further investigation.

## 2. Results

### 2.1. Study Population—Clinicalpathological Features

The main clinicopathological features of the present ccRCC series are summarized in Table 1. There were slightly more male patients than females, with a mean age of 58 years old (ranging from 20 to 81 years). More than half of the patients were white and presented localized disease (stages I and II). Approximately 20% of the ccRCC patients had metastasis at diagnosis and received systemic therapy. The mean follow-up time was four years and almost 70% of the patients were alive without disease. Disease progression and cancer-specific death was seen in 19.5% of the cases (Table 1). The major risk factors for ccRCC seen in this cohort were hypertension (54.3%), smoking (33.6%) and diabetes (22.8%) (Table S1). The 5-year overall survival rate for ccRCC cancer patients was 77.3%, with an estimated median survival time of 53.5 months. Kaplan–Meier plots showed that OS was significantly lower for patients in advanced stages (*p* = 0.01) and higher histopathological grade (*p* < 0.001), metastasis at diagnosis (*p* = 0.002), and tumors larger than 7 centimeters (*p* = 0.003) (Table S1, Figure S1 and Figure S2).

### 2.2. Analysis of Somatic Copy Number Alterations and Integration with In Silico Transcriptomic Profile

**Figure 1:**
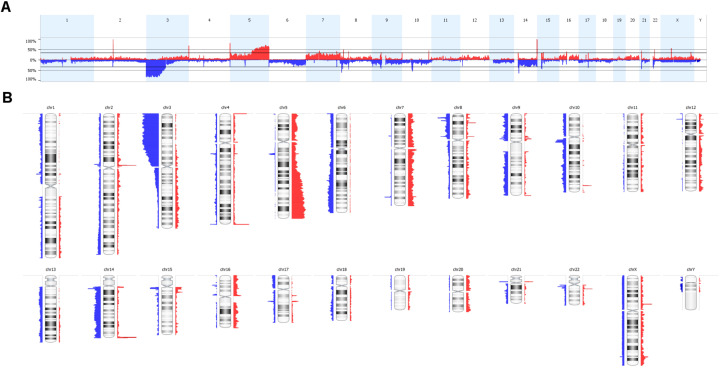
Somatic DNA copy number aberrations (CNAs) of 92 patients of ccRCC. In (**A**) The linear distribution of DNA gains (red) and losses (blue) and in (**B**) Chromosomal representation of all the aberrations.

The somatic CNA landscape is shown in Figure 1. A median of 67.5 alterations per patient tumor was found. An average of 10–12% of each patient’s tumor genome had copy number imbalances. STAC analysis identified 158 regions of interest (Table S2). Most significant regions included those related to loss of 3p (87.3%), 14q (35.8%), 9p (28.6%) and 10q (25.0%), and gains of 5q (59.7%), 6q (29.3%), 7p (29.3%) and 16q (20.6%) (Figure 1).

Bioinformatics analysis using Nexus Copy Number software disclosed 19 genes mapped to CNA significant regions (Table 2). Compared to TCGA data, there was a modest difference in overall CNA frequencies.

**Figure 2:**
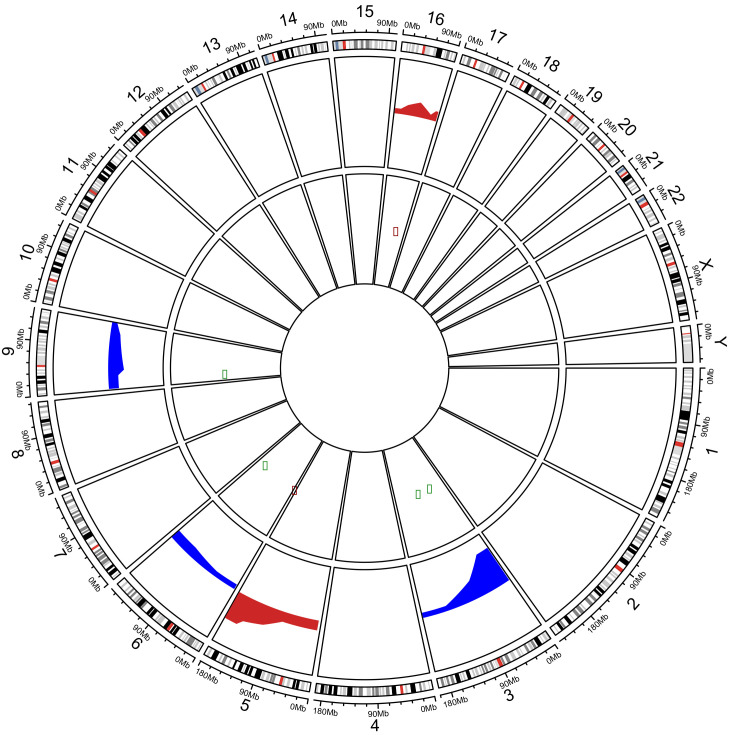
Circos Plot showing the correlation of CNAs and gene expression from Oncomine data. The periphery circle represents regions of Copy Number Aberrations along the genome per chromosome. Red represents copy number gains and blue copy number losses. The inner circle contains genes with concomitant alteration in gene expression. Green dots represents down-regulated genes and red up-regulated genes.

According to Cancer Genome Interpreter (CGI) analysis, alterations in *SETD2*, *BAP1*, *FLT4* and *PTEN* genes were reported as known drivers in several tumor types, and events in *FGFR4* and *NSD1* genes were classified as predicted drivers. Next, to look for putative correlations between CNAs and gene’s expression profile, the 19 genes were investigated for gains or losses of expression in TCGA dataset through the Oncomine platform (504 ccRCC samples) (504 ccRCC samples). Losses of *BAP1*, *MYB*, *MLLT3* and *TFG* showed concurrent underexpression. Likewise, gains of *CDH11* and *FLT4* genes showed overexpression in TCGA dataset (Figure 2).

### 2.3. CNAs and Gene Expression Clinical Impact and Survival Analysis

The significant regions mentioned above were further investigated for their clinical associations (Table 3). Except for 3p loss, all other alterations, i.e., deletions on 9p (9p-) and 14q (14q-), and gain on 7p (7p+) were significantly associated with features of advanced disease, such as tumor metastasis, T3/T4 stages, higher Fuhrman grade (3–4), larger tumors and dismal outcome (death).

The multivariate analysis showed that the gain in the 5q has been shown to increase the risk of large tumors (over 7 centimeters) (*p* = 0.03; 95% CI = 1.08–8.14). Patients with a gain of 7p21.2 are more likely to be diagnosed with tumors that affect adjacent structures (T3 and T4) (*p* = 0.02; 95% CI = 1.11–8.04) and in advanced histological degrees (*p* = 0.01; 95% CI = 1.28–9.10). Patients with loss of 9p21.3 are more likely to have advanced staging (*p* = 0.005; 95% CI = 1.51–10.20) and with metastatic disease (*p* = 0.003; 95% IC = 1.72–16.02). Finally, patients with a loss of 14q22.1 had an increased risk of death approximately 3 times compared to patients without this event (*p* = 0.02; 95% CI = 1.12–7.84) (Table 4).

**Figure 3:**
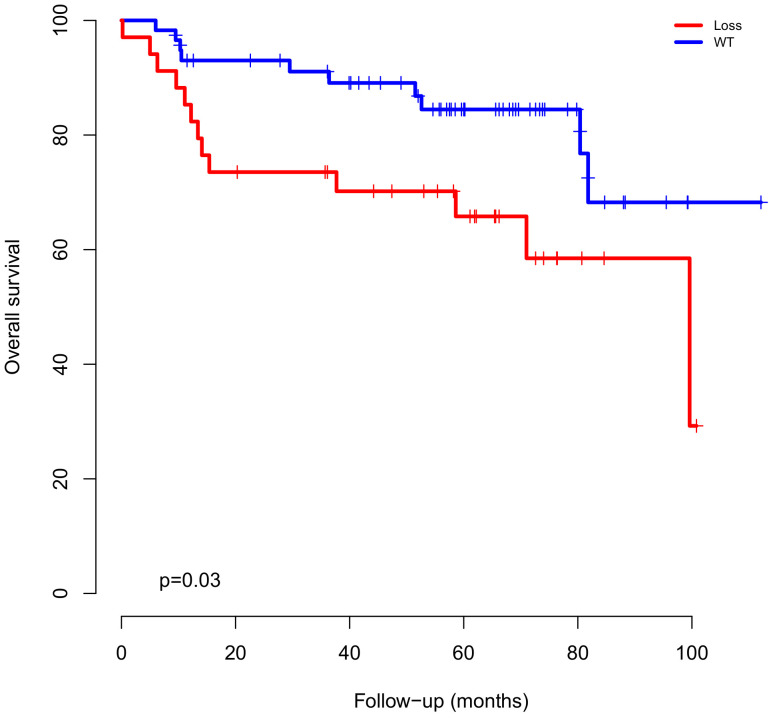
Overall survival of chromosome 14 q 22.1 which encompasses the *NIN* gene.

In addition, loss of chromosome 14q, which encompasses the *NIN* gene was associated with shorter survival (*p* = 0.03) (Figure 3). Interestingly, 5q gain (5q+) was also associated with poor outcomes (Table 3).

We further investigated the putative associations between CNA burden (total number of CNAs and PGA) and clinicopathological features (Table 5). Patients harboring tumors with more than 50 CN alterations (25th percentile) presented larger tumor size (*p* = 0.002), poor outcome (death) (*p* = 0.04) and higher Fuhrman grades (*p* = 0.01) in the multivariate analysis (Table 5). However, when using PGA for multivariate analysis, it was not significative, i.e., tumor size (≥7 cm), outcome (death) and Fuhrman grade showed *p* = 0.08, *p* = 0.33 and *p* = 0.13, respectively.

We next investigated whether some genes of interest that were disclosed in our study could be also altered in the TCGA dataset through Oncomine platform (Table 6). According to TCGA data, loss of *BAP1* in 3p, an oncogenic aberration known to be a biomarker of RCC was only associated with Fuhrman grade 1 (*p* = 0.02). Losses of *MYB* and *MLLT3*, despite being predicted as passengers alterations were significantly associated with advanced disease: Loss of *MLLT3* gene was associated with metastasis at diagnosis (*p* = 0.005), stage IV disease (*p* = 0.02), larger tumors (*p* = 0.01) and poor outcomes (*p* = 0.02), while loss of *MYB* was correlated with Fuhrman grade 3 (*p* = 0.02). Additionally, gains on 5q genes showed significant association with increased cancer aggressiveness. Loss of *FTL4* was also associated with larger tumors (*p* = 0.007) and loss of *NSD1*, considered a predicted driver aberration was associated with larger tumors (*p* = 0.007) and advanced disease (stage IV; *p* = 0.01). Aberrations of *TFG* and *CDH11*, despite showing changes on gene expression levels are still reported as predicted passenger alterations and did not show any clinicopathological associations.

## 3. Discussion

Copy number alterations (CNA) are important drivers of ccRCC tumorigenesis [4]. This study provides the first comprehensive report of CNA profiles in Brazilian ccRCC patients. We observed significant numbers of CNA, including loss of 3p (87.3%), 14q (35.8%), 6q (29.3%), 9p (28.6%) and 10q (25.0%), and gains of 5q (59.7%), 7p (29.3%) and 16q (20.6%). Some regions, including the gain of 5q34–q35.3 (*FLT4* and *NSD1*) and loss of 6q23.2–q23.3 (*MYB*), 9p21.3 (*MLLT3*), and 14q22.1 (*NIN*) had gene expression levels validated by in silico re-analysis of TCGA data and were associated with patients clinicopathologic features.

Overall, the frequency of CNA in chromosomal arms and whole chromosomes reveal similar profiles to those demonstrated by others, especially the TCGA [10]. We then re-analyzed TCGA aCGH array data with the same analysis parameter settings used for Brazilian patients. The CNA profile in Brazilian patients differs from TCGA only for chromosomes 6, 9, and 10. A possible explanation is that tumor events take place over time, and some important drivers, in general, occur at the beginning of the carcinogenesis process [24]. It has been reported that 3p loss is a truncal event in RCC [25,26]. Furthermore, the gain of 5q and loss of 14q are usually shared by different patient cohorts and are likely clonal events that occur early in the ccRCC disease process [25,26]. In our data, both changes are recurrent and similar to TCGA. Despite few differences, variations across populations have been reported in some cancer types. Recently, variations in *VHL* and *PBRM1* mutation rates were observed according to ancestry, with fewer mutations in African kidney cancer patients [27]. Moreover, African American kidney cancer types from TCGA data harbor a lower level of chromosomal instability [28]. Further studies on ccRCC mutations in the Brazilian population and local ancestry inference are necessary to estimates the number of copies at genomic sites to unravel such differences. Additionally, loss of 3p in our patient cohort was similar between metastatic and non-metastatic tumors (88% vs. 84%, respectively). Although it is associated with initial histological grade by univariate analysis, there was an absence of multivariate significance and no correlation with survival. Since most of the samples in our study are primary tumors of clinical stages I/II, lower frequencies of chromosome imbalance would be expected. One limitation of our cohort is that it may not be possible to identify relevant aberrations specifically related to metastatic disease or advanced disease.

Gunawan and colleagues suggest that a gain of 5q is a clinically favorable alteration in ccRCC with an increase in overall survival rate [17]. Moreover, gains of 5q were further reported with a frequency twice as high in non-metastatic tumors than in metastatic ones [29]. However, 5q gain was not associated with significant differences in overall survival in our cohort.

Furthermore, the 14q loss, associated with loss of *HIF1A*, is another recurrent alteration in ccRCC and has been predicted to drive more aggressive disease [10]. Several studies showed a significant correlation of alterations in this region with a worse prognosis, distant metastasis and decreased overall survival in accordance with our findings [16,21,30,31]. It was also demonstrated that the loss of chromosome 14 leads to a decrease in HIF1-a protein levels since *HIF1A* gene maps at 14q23.2 [30]. The 14q22.1 region which encompasses the *NIN* gene was associated with decreased overall survival and was also identified in our analysis of the TCGA data. In our findings, 77% of patients with loss of *NIN* also showed concomitant loss of *HIF1A*. The *NIN* gene has been initially described as playing a role to centrosomal function [32]. The involvement of this gene in cancer is unclear. Future studies are warranted to clarify the role of *NIN* gene in the ccRCC tumorigenesis.

Concerning additional cancer genes identified in the significantly altered regions, some were characterized as known drivers (*SETD2*, *BAP1*, *FLT4* and *PTEN*) and others as predicted drivers (*FGFR4* and *NSD1*), according to the CGI. The CGI tool identifies as driver genes according to their mode of action in tumorigenesis (i.e., whether they function as oncogenes or tumor suppressors), based on either experimentally verified sources or in silico prediction [33]. However, despite the losses of *MYB* and *MLLT3* were considered to be predicted passenger by CGI analysis, our findings show concordance with gene expression and association with advanced disease. Another important known driver is the loss *PBRM1*, found in 83% of our cohort despite not being identified in significant regions by the analysis of STAC. Deletions of *PTEN*, *SETD2* and *BAP1* genes, are described as biomarkers for RCC and are responsive to drugs of preclinical and clinical trials [34,35,36,37,38,39]. Also, deletions of *JAK2*, *CD274* and *MYD88* genes, and amplification of *FGFR4* and *NPM1* genes, are also involved in clinical trials for other tumor types [40,41,42,43]. Interestingly, alterations in *JAK2*, *MYD88* and *NPM1* genes, already have drugs FDA approved for other cancers [44,45,46]. Additionally, the loss of *NSD1* gene, a possible driver alteration associated with tumors ≥7 cm and advanced stages. *NSD1* gene located at chromosome 5, which encodes for the nuclear receptor-binding SET-domain containing protein 1 (NSD1). Little is known regarding the function of *NSD1*, but its genomic location is near *FGFR4*. Since the *NSD1* gene alteration was not validated *in silico* in our gene expression analysis, it is possible that other genes in the region could play a driver role in such region in ccRCC.

Altogether, it is essential to consider that changes in CNAs could not affect gene expression levels due to transcriptional adaptative mechanisms. A recent study used a new method, known as transcriptional adaptation to CNA profiling (TACNA), and found a strong correlation of expression levels changes by CNAs with mean methylation from TCGA datasets [47]. These findings suggest that dysregulation of the epigenome could be one of the underlying mechanisms driving the translational response to CNAs, and further studies could help to understand the impact of the results regarding tumor suppressors involved in the chromatin modification mechanisms. Herein, we also addressed the poorly explored relationship between the overall CNA load and clinical data in ccRCC. Arai and colleagues in an unsupervised hierarchical cluster analysis based on aCGH results were also able to separate patients into 2 groups according to the CNA pattern [48]. In our current study, the group of patients with less than 50 CNAs was significantly associated with evidence of localized diseases such as small tumors, early stages and histological grade, absence of metastases, and better outcomes. Increased CNAs has been reported as indicative of chromosomal instability and have been associated with poor cancer outcome [49]. Despite a high number of studies associating tumor mutation burden with immune cell infiltrates, studies reporting the impact of CNA load in predict the response to targeted therapies and the outcome are scarce. Identifying biomarkers could better select treatments and improve survival in patients with metastatic ccRCC and CNAs could provide new approaches for identifying potential subtypes and pathways suitable for targeted therapies and improved outcome.

## 4. Materials and Methods

### 4.1. Sample Collection

This study comprises a retrospective cohort of 92 treatment-naïve patients diagnosed with clear-cell renal-cell carcinoma (ccRCC) that underwent nephrectomy between 2008 and 2014 at Barretos Cancer Hospital. All clinical data were obtained from medical records and managed through RedCap Research Electronic Data Capture (Vanderbilt University, Nashville, TN, USA) (https://hcbredcap.com.br). The study protocol was approved by the Internal Review Board (IRB No. #8042014). Tumor samples were dissected from surgical specimens, snap-frozen in liquid nitrogen and stored at −80 ${}^{\circ }$C until DNA preparation. Histopathological evaluation was performed independently by two pathologists (CSN and LN) according to the last WHO classification system [50]. Nuclear grade was scored according to the Fuhrman classification system [51]. Patients without histologically confirmation or non-classic ccRCC histopathology were excluded.

### 4.2. DNA Isolation

Tumor DNA was isolated from frozen tissue with >60% tumor cellularity and necrosis was <20%. DNA isolation was performed using QIAsymphony DNA mini Kit following the manufacturer’s instructions. DNA quality and integrity were assessed by NanoDrop and quantified by Qubit® Fluorometric Quantitation (Life Technologies). Samples with insufficient material for research or of DNA poor quality were removed from the analysis.

### 4.3. DNA Labeling and Hybridization

CGH-array was performed according to the standard protocol from Agilent SurePrint G3 CGH+SNP 4 × 180 K (Agilent Technologies, Santa Clara, CA, USA) using a diploid DNA reference as control (Agilent Technologies). Normal DNA (reference) was labeled with Cyanine-3 and tumor DNA (test) with Cyanine-5 (Agilent Technologies). Labeled DNAs were purified, and the quality of labeling was assessed by NanoDrop (Thermo Fisher Scientific, Waltham, MA, USA). Equal quantities of DNA (test and reference) from each patient were hybridized into the slides for 24 h and cleaned with washed and stabilization buffers, plus acetonitrile. The slides were scanned using a SureScan Microarray Scanner (Agilent Technologies, Santa Clara, CA, USA) following the manufacturer’s recommendations and as previously reported by our group [52]. The recommended cut-off Derivative of the Log Ratio Spread (DLRS) considered for the aCGH experiments was 0.30.

### 4.4. Bioinformatics and Statistical Analysis

CNA analysis was performed using Nexus Copy Number software (BioDiscovery Inc., El Segundo, CA, USA) (http://www.biodiscovery.com/nexus-copy-number/). In this study, we used the definition that CNA (Copy Number Alteration) refers to somatic events. BioDiscovery’s SNP-FASST2 Segmentation Algorithm, is an extension of the FASST2 Segmentation Algorithm (a Hidden Markov Model [HMM]-based approach that unlike other common HMM methods does not aim to estimate the copy number state at each probe but uses many states to cover more possibilities, such as mosaic events, and then make calls based on a second level threshold). With the SNP-FASST2 algorithm, B-allele frequency probes are assigned to a range of possible states and a combination of the BAF and Log-R states are used to make the final copy number and allelic event calls. The recommended parameters for Agilent CGH+SNP arrays were: selection of ProcessedSignal from Agilent Feature Extraction (FE) software (Agilent Technologies), exclusion of saturated (IsStaturated) and non-uniform (IsFeatNonUnifOl) spots as flagged from FE, the significance threshold for segmentation was set at 5.0 × 10${}^{-6}$ also requiring a minimum of 3 probes per segment. The log ratio thresholds for single copy gain and single copy loss were set at 0.18 and −0.18, respectively. The log ratio thresholds for two or more copy gain and homozygous loss were set at 0.7 and −1.1 respectively. The Homozygous Frequency Threshold was set to 0.85. The Homozygous Value Threshold was set to 0.75. The Heterozygous Imbalance Threshold was set to 0.3. The minimum length was set at 1000 base pairs (bp). A 3:1 sex chromosome gain threshold was set to 1.2 and a 4:1 sex chromosome gain threshold was set to 1.7. Male sex chromosome big loss threshold was set to −1.1. The Significance Testing for Aberrant Copy number (STAC) [53], which is based on global frequency statistic approach, was applied to identify regions of the genome with a statistically high frequency of aberration. It was considered a *p*-value ≤ 0.05 with 1000 permutations as input parameter, as well as the aggregate cut-off filter of ≥ 20%. An additional filter was applied to regions showing 100% overlap with previously identified CNVs from the Database of Genomic Variants (DGV), inside the Nexus Copy Number platform. The exception were regions with somatic cancer genes identified by Cancer Gene Census. Significant regions were queried for oncogenic alterations through Cancer Genome Interpreter platform (CGI) (http://www.cancergenomeinterpreter.org). The threshold value used to stratify the CNA burden was within the 25th percentile, accounting for approximately 50 alterations, similarly to previous studies [54,55]. We also used the percentage of genome alteration (PGA) in the analysis. The Oncomine™ transcriptomic cancer profiling platform (Compendia Bioscience, Ann Arbor, MI, USA) (https://www.oncomine.org/) was used to explore changes in gene expression of the regions found. The premium version of Oncomine™ has currently 715 independent datasets and 86,733 samples normalized by the same parameters, including the TCGA data. Genes were investigated for gains and losses of expression in TCGA dataset (totaling 579 ccRCC clinical samples with DNA copy number data) [10,56]. The R Package for statistical analysis (https://www.R-project.org) was locally implemented in Galaxy platform (https://usegalaxy.org) for the associations. Correlation between frequencies of molecular alterations and clinical, histological and pathological features were performed using chi-square or Fisher’s exact test, both one-sided and two-sided tests, with a significance set at *p*≤ 0.05. The *p*-values were adjusted according to the Benjamini–Hochberg false-discovery (FDR) method using the p.adjust function in R (https://www.R-project.org). For this analysis, it was used the 12 regions identified by STAC and the following clinical features (Fuhrman grade, metastasis, tumor size, tumors T3–T4, clinical stage and death). The multivariate analysis by the Binary Logistic Regression method was performed using the Backward model with variables with significance equal to or below 0.2 after FDR correction in the univariate analysis. The IBM SPSS Statistics software version 23 (https://www.ibm.com/analytics/spss-statistics-software) was used to perform the multivariate and survival analysis. Overall survival (OS) was defined as the time from nephrectomy until the last contact or death. OS was assessed using the Kaplan–Meier method and log-rank comparisons, considering *p*≤ 0.05 as the significance value.

## 5. Conclusions

In summary, we report the first CNA landscape of Brazilian ccRCC. We found the most frequent aberrant regions were loss of 3p (87.3%), 14q (35.8%), and gains of 5q (59.7%) in accordance with previous studies. We identified that CNAs in 5q34–q35.3 (*FLT4* and *NSD1*), 6q23.2–q23.3 (*MYB*), 9p21.3 (*MLLT3*) and 14q22.1 (*NIN*) associated with patient’s aggressive disease and identified putative CNA-associated genes. This study can pave the way to the understand ccRCC biology and the CNA biomarkers in Brazilian patients.

## Figures and Tables

**Table 1:** Patient clinicopathological features.

Clinical Data	Category	Patients (Frequency)
**Gender**	Male	50 (54.3%)
	Female	42 (45.7%)
	Alive	69 (75.0%)
**Status follow-up**	Deceased	23 (25.0%)
**Age**	Average (min-max)	58 (20–81)
	I/II	60 (65.2%)
**Clinical stage**	III/IV	32 (44.8%)
**T**	T1a-b	47 (51.1%)
	T2a-b	23 (25.0%)
	T3a-c	17 (18.5%)
	T4	5 (5.4%)
	N0	77 (83.7%)
	N1	9 (9.8%)
**N**	Nx	6 (6.5%)
**M**	M0	75 (81.5%)
	M1	17 (18.5%)
	<7 cm	58 (63.0%)
**Tumor size**	≥7 cm	34 (37.0%)
**Furhman Grade**	1	20 (21.7%)
	2	49 (53.3%)
	3	16 (17.4%)
	4	7 (7.6%)
	White	64 (69.6%)
	Black	3 (3.3%)
	Yellow	1 (1.0%)
	Brown *	20 (21.7%)
**Self-Declared Ethnicity**	Unknown	4 (4.4%)
**Systemic Therapy**	Sunitinib	12 (13.0%)
	Interferon	4 (4.3%)
	Pazopanib	1 (1.1%)
	None	74 (80.4%)
	Other	1 (1.1%)
	Alive without disease	64 (69.6%)
	Alive with disease progression	2 (2.1%)
	Progression and death from the disease	18 (19.6%)
**Disease status (last follow-up)**	Died without disease recurrence	8 (8.7%)

* Brown = Brazilian mixed; T = size or direct extent of the primary tumor; N = degree of spread to regional. lymph nodes; M = presence of distant metastasis.

**Table 2:** Frequency of significant regions according to STAC with frequency ≥20% in the present casuistic and in TCGA.

Chromosome	Cytoband	Event	Patients Frequency (%)	TCGA Frequency (%)	Cancer Genes
Chr3	p21.2–p21.3	DEL	83.7	72.2	*BAP1*, *SETD2*
Chr3	p22.2	DEL	83.7	72.2	*MYD88*
Chr3	p25.2	DEL	83.7	72.2	*PPARG*
Chr3	q11.2-q13.11	DEL	20.6	25.7	*TFG*, *CBLB*
Chr5	q34-q35.3	AMP	59.7	47.6	*RANBP17*, *NPM1*, *FGFR4*, *NSD1*, *FLT4*
Chr6	q23.2–q23.3	DEL	29.3	19.0	*MYB*
Chr7	p21.1	AMP	29.3	22.9	*ETV1*
Chr9	p21.3	DEL	28.2	17.9	*MLLT3*
Chr9	p24.1	DEL	27.1	20.7	*JAK2*, *CD274*
Chr10	q23.31	DEL	25.0	13.6	*PTEN*
Chr14	q22.1	DEL	35.9	29.0	*NIN*
Chr16	q21	AMP	20.6	11.9	*CDH11*

AMP = Amplification; DEL = Deletion.

**Table 3:** Association of clinical features with significant regions identified by STAC.

Chromosome	Cytoband	Event	Clinical Features	*p* Value	FDR
Chr3	p21.2	DEL	Fuhrman Grade (1-2)	0.02	0.15
Chr3	p25.2	DEL	Fuhrman Grade (1–2)	0.01	0.14
Chr5	q34–q35.1	AMP	Metastasis	0.03	0.18
			Tumor size (≥7 cm)	0.01	0.14
			Fuhrman Grade (3–4)	0.01	0.14
Chr7	p21.2	AMP	Tumors T3/T4	0.02	0.15
Chr9	p21.3	DEL	Metastasis	0.01	0.14
			Tumor size (≥7 cm)	0.02	0.15
			Tumors T3–T4	0.04	0.19
			Clinical stage (3–4)	0.01	0.14
			Tumor size (≥7 cm)	0.04	0.19
Chr9	p24.1	DEL	Tumors T3-T4	0.04	0.19
Chr14	q22.1	DEL	Clinical stage (3–4)	0.04	0.19
			Outcome (death)	0.02	0.15
			Metastasis	0.03	0.19
			Fuhrman Grade (3–4)	0.01	0.14

AMP = Amplification; DEL = Deletion.

**Table 4:** Multivariate analysis of clinical features associated with STAC significant regions.

Clinical Feature	Region	RR	95% CI	*p* Value
Tumors T3/T4	7p21.2 (AMP)	3.0	1.11–8.04	0.02
Tumor size (>7 cm)	5q35.2–q35.3 (AMP)	2.9	1.08–8.14	0.03
	9p24.1 (DEL)	3.3	1.25–9.04	0.01
Clinical Stage (3–4)	9p21.3 (DEL)	3.9	1.51–10.20	0.005
Outcome (Death)	14q22.1 (DEL)	2.9	1.12–7.84	0.02
Fuhrman Grade (3–4)	7p21.2 (AMP)	3.4	1.28–9.10	0.01
Metastasis	9p21.3 (DEL)	5.2	1.72–16.02	0.003

AMP = Amplification; DEL = Deletion; RR = Relative Risk; CI = Confidence interval.

**Table 5:** Multivariate analysis of CNA burden.

Clinical Feature	Category	RR	95% CI	*p* Value
Tumor size (≥7 cm)	> 50 CNA	7.8	2.14–28.55	0.002
Outcome (death)	> 50 CNA	3.7	1.02–14.02	0.04
Fuhrman Grade (3–4)	> 50 CNA	14.1	1.80–111.13	0.01

RR = Relative Risk; CI = Confidence interval.

**Table 6:** Enrichment analysis for genes associated with gene expression through Oncomine platform.

Chr	Cytoband	Gene	CN Event	Expression	Clinical Features	*p*-Value
3	p21.2–p21.3	BAP1	Loss	Downregulation	Fuhrman Grade (1–2)	0.02
5	q34–q35.3	FLT4	Gain	Upregulation	Tumor size (>7 cm)	0.007
5	q34–q35.3	NSD1	Gain	Upregulation	Tumor size (>7 cm)	0.007
					Clinical Stage (3-4)	0.01
6	q23.2–q23.3	MYB	Loss	Downregulation	Fuhrman Grade (3-4)	0.02
9	p21.3	MLLT3	Loss	Downregulation	Metastasis	0.005
					Clinical Stage (3–4)	0.02
					Tumor size (>7 cm)	0.01
					Outcome (death)	0.02
					Fuhrman Grade (3–4)	0.01

## Data Availability

Data is available from the corresponding authors on reasonable request.

## Supplementary Materials

The following are available at https://www.mdpi.com/1422-0067/22/5/2265/s1. Table S1: Summary of the main risk factors associated with ccRCC patients; Table S2: All 158 regions of interest identified by STAC analysis; Figure S1: Overall survival of the 92 ccRCC patients; Figure S2: Kaplan Meier plots according to stage (A), histological grade (B), metastasis (C) and tumor size (D).
