# Supplementary material for: Somatic Copy Number Alterations and Associated Genes in Clear-Cell Renal-Cell Carcinoma in Brazilian Patients
Source: Int J Mol Sci. 2021 Feb 25;22(5):2265. doi: 10.3390/ijms22052265 (PMC7956176; doi:10.3390/ijms22052265)
Supplement: Supplementary file 1 [file ijms-22-02265-s001.zip › Supplementary ok/supplementary_corrected.pdf]

Supplementary Materials: Somatic Copy Number Alterations and Associated Genes in Clear-Cell Renal-Cell Carcinoma in Brazilian Patients

Flávia Gonçalves Fernandes[1], Henrique Cesar Santejo Silveira[1], João Neif Antonio Júnior[2], Rosana Antunes da Silveira[1], Luis Eduardo Zucca[2], Flavio Mavignier Cárcano[2,3], André Octavio Nicolau Sanches[2], Luciano Neder[4], Cristovam Scapulatempo-Neto[4], Sergio Vicente Serrano[2,3], Eric Jonasch[5], Rui Manuel Reis [1,6,7*] and Adriane Feijó Evangelista [1*]

Table S1. Summary of the main risk factors associated with ccRCC patients.

Clinical Data	Category	Patients (Frequency)
Obesity	Yes	31 (33.6%)
	No	61 (66.3%)
Smoke	Yes	14 (15.2%)
	No	56 (60.8%)
	Former smoker	17 (18.4%)
	Unknown	5 (5.4%)
Alcohol	Yes	19 (20.6%)
	No	67 (72.8%)
	Unknown	6 (6.5%)
Conditions	Diabetes	21 (22.8%)
	Hypertension	50 (54.3%)
	Diabetes and Hypertension	20 (21.7%)
	Other	8 (8.6%)

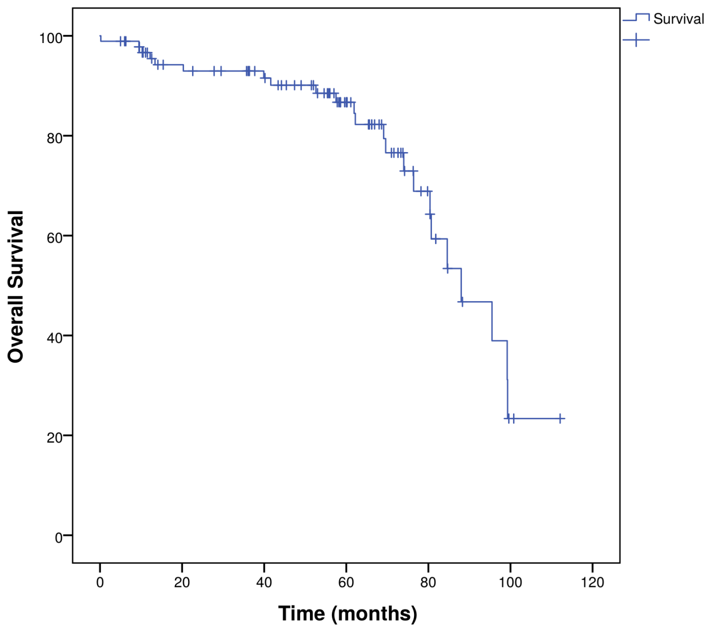

Figure S1. Overall survival of the 92 ccRCC patients.

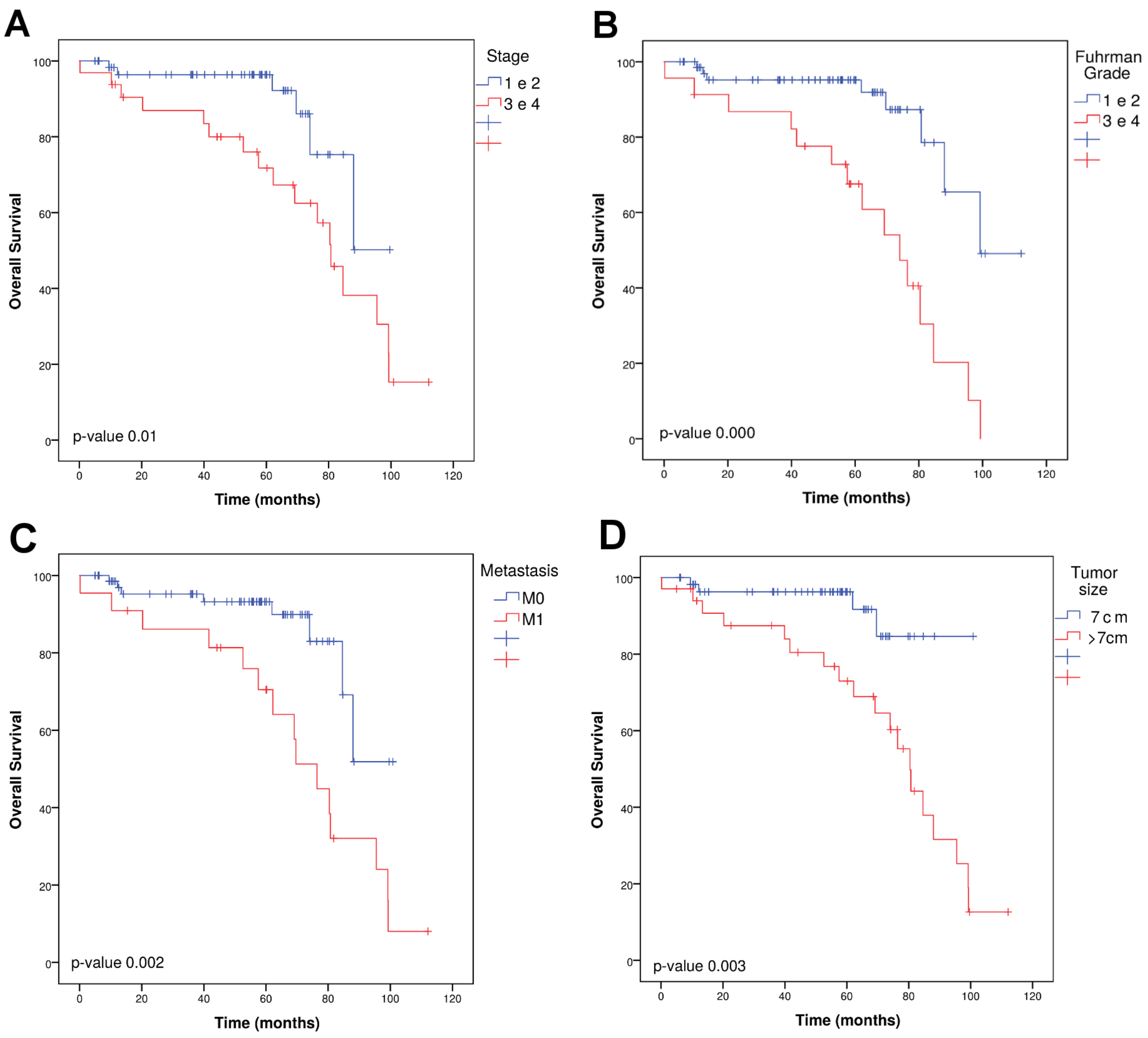

Figure S2. Kaplan Meier plots according to stage (A), histological grade (B), metastasis (C) and tumor size (D).
